# Longitudinal changes in body composition and waist circumference by self-reported levels of physical activity in leisure among adolescents: the Tromsø study, Fit Futures

**DOI:** 10.1186/s13102-019-0150-8

**Published:** 2019-12-17

**Authors:** Nils Abel Aars, Bjarne K. Jacobsen, Bente Morseth, Nina Emaus, Sameline Grimsgaard

**Affiliations:** 1 Department of Community Medicine, UiT The Arctic University of Norway, 9037 Tromsø, Norway; 2 Nordland Hospital, Bodø, Norway; 3 Centre for Sami Health Research, Department of Community Medicine, UiT The Arctic University of Norway, Tromsø, Norway; 4 School of Sport Sciences, UiT The Arctic University of Norway, Tromsø, Norway; 5 Department of Health and Care Sciences, UiT The Arctic University of Norway, Tromsø, Norway

**Keywords:** Adolescence, Body composition, Longitudinal study, Physical activity

## Abstract

**Background:**

It is not clear how physical activity affects body composition in adolescents. Physical activity levels are often reduced during this period, and the relative proportion of body fat mass and lean mass undergo natural changes in growing adolescents. We aimed to examine whether self-reported physical activity in leisure time at baseline or change in activity during follow-up affect changes in four measures of body composition; body mass index (kg/m^2^), waist circumference, fat mass index (fat mass in kg/m^2^) and lean mass index (lean mass in kg/m^2^).

**Methods:**

We used data from the Tromsø Study Fit Futures, which invited all first year students in upper secondary high school in two municipalities in northern Norway in 2010–2011. They were reexamined in 2012–2013. Longitudinal data was available for 292 boys and 354 girls. We used multiple linear regression analyses to assess whether self-reported level of physical activity in leisure time at baseline predicted changes in body composition, and analysis of covariance to assess the effects of change in level of activity during follow-up on change in body composition. All analyses were performed sex-specific, and a *p*-value of < 0.05 was considered statistically significant.

**Results:**

There were no associations between self-reported leisure time physical activity in the first year of upper secondary high school and changes in any of the considered measure of body composition after 2 years of follow up, with the exception of waist circumference in boys (*p* = 0.05). In boys, change in fat mass index differed significantly between groups of activity change (*p* < 0.01), with boys adopting activity or remaining physically active having less increase in fat mass index than the consistently inactive. In girls, change in lean mass index differed significantly between groups of activity change (*p* = 0.04), with girls adopting physical activity having the highest increase.

**Conclusions:**

Self-reported leisure time physical activity does not predict changes in body composition in adolescents after 2 years of follow up. Change in the level of physical activity is associated with change in fat mass index in boys and lean mass index in girls.

## Background

Overweight or obesity in adolescence is a major risk factor for the same conditions as an adult [1], and therefore a risk factor for cardiovascular disease, type II diabetes, several types of cancer and musculoskeletal disorders in adulthood [2]. More than 20% of adolescents in Norway were in 2010 classified as overweight or obese [3]. Among adolescents in the Western world there is evidence for a plateauing of the obesity epidemic at a high level [4]. In Norwegian men and women, the prevalence of both overweight and obesity is increasing [5–7]. Studies have shown that adolescent lifestyle tend to persist into adulthood [8, 9], emphasizing the importance of preventing overweight and obesity in this period of life. A systematic review on the relationship between body composition and physical activity in adolescents showed conflicting results, with reverse causality suggested as a possible explanation – meaning that overweight and obesity could be both a cause and an effect of low physical activity [10]. The relationship is further complicated by associations with sedentary behavior, nutrition, socio-economic status and genetics [11–14].

There are several ways to quantify physical activity in adolescents. The use of direct observation of individuals or doubly labelled water has been suggested as gold standards, but questionnaire data are more feasible, domain specific and common in observational studies [10]. However, it must be acknowledged that self-reported physical activity tends to exaggerate the true amount of physical activity when compared to data from, for instance, accelerometers [15].

Arguably, the most common measure of body composition is body mass index (BMI, body weight in kg/m^2^), but its ability to identify changes in adiposity is limited as it does not distinguish between changes in fat mass and changes in lean mass [16]. This is a challenge when studying body composition in growing adolescents because boys naturally tend to gain more muscle mass than girls, while girls naturally gain more fat mass [17]. In addition to BMI, we therefore included waist circumference, fat mass index (FMI, fat mass in kg/m^2^) and lean mass index (LMI, lean mass in kg/m^2^) as measures of body composition in the present study. Waist circumference is an anthropometric measure which is specific to abdominal fatness [18]. FMI and LMI has been advocated as good measures of changes in adiposity in longitudinal studies because they measure fat mass and lean mass in relation to height [17, 19]. There are few studies investigating the longitudinal association between self-reported physical activity and tissue specific measures of body composition in adolescents [10], with a majority of those available using BMI as the primary outcome. To our knowledge, no studies modelling the association between physical activity and changes in FMI or LMI have been performed in Norway. Some international evidence points to a positive association between physical activity over the course of adolescence and LMI at age 18, but a less clear relationship with FMI [20]. Furthermore, higher self-reported physical activity has been associated with a positive change in lean mass [21], but not in fat mass [22].

We examined whether self-reported physical activity during leisure time was associated with change in measures of body composition after 2 years in upper secondary school in a cohort of adolescents in northern Norway; from a first measurement in 2010–2011 to a second measurement in 2012–2013. We further investigated whether changes in body composition differ between adolescents who are persistently inactive, persistently active, adopt activity or quit activity over the same period.

## Methods

The Tromsø Study Fit Futures is a population-based cohort study, conducted in 2010–2011 (Fit Futures 1) and repeated in 2012–2013 (Fit Futures 2). The study invited all students in their first (Fit Futures 1) and third (Fit Futures 2) year of upper secondary school in the neighboring municipalities of Tromsø and Balsfjord in northern Norway. Fit Futures 1 invited 1117 students, with 1038 (93%) attending. Fit Futures 2 invited 1130 students and 870 (77%) attended. The participants in both studies answered a questionnaire and underwent a clinical examination at the clinical research unit at the University Hospital in Northern Norway, as detailed previously [23]. The present study includes only those participating in both Fit Futures 1 and Fit Futures 2. We excluded participants aged 18 years or older at baseline (Fit Futures 1), those without valid measurements of BMI, waist circumference, FMI and LMI at baseline and follow-up, and participants without information on physical activity at baseline. Altogether 292 boys and 354 girls were eligible for analyses.

Body weight was measured to the nearest 100 g with light clothing and height was measured to the nearest 0.1 cm on a Jenix DS 102 automatic electronic scale/stadiometer (Dong Sahn Jenix, Seoul, Korea). Waist circumference was measured to the nearest cm after expiration and at the height of the umbilicus. Total body fat mass and total body lean mass was measured using GE Lunar Prodigy dual-energy x-ray absorptiometry scanner (Lunar Corporation, Madison, Wisconsin, USA). Lean mass is comprised of all bodily tissue except fat and bone. Based on these measurements, Fat Mass Index (FMI, fat in kg/height in meters^2^) and Lean Mass Index (LMI, lean mass in kg/height in meters^2^) was calculated.

The prevalence of overweight or obesity in Fit Futures 1 was determined by applying the International Obesity Task Force body mass index reference values for adolescent populations, using age in half years [24, 25]. The participants were classified as underweight, normal weight, overweight or obese. These reference values correspond to an adult (aged 18 and above) BMI of < 18.5 kg/m^2^, 18.5 ≤ BMI < 25 kg/m^2^, 25.0 ≤ BMI < 30 kg/m^2^, and BMI ≥ 30.0 kg/m^2^, respectively.

The outcomes in this study were change in BMI, waist circumference, FMI and LMI between Fit Futures 1 and Fit Futures 2. The other variables included in the analyses were derived from the questionnaires. Our primary exposure was self-reported physical activity in leisure time, measured using the question “*Are you physically active outside school hours? Yes/no”*. Those answering “*No*” were labelled as physically inactive. Those answering “*Yes*” were asked “*How many hours per week are you physically active outside of school hours?”*. This question was used in the Health Behavior in School Children study and was validated for an adolescent population [26]. There are six response categories, from none to more than 7 h per week. One person in Fit Futures 1 reported “*none”* on this question, and was therefore also labelled as physically inactive. “*About half an hour*” and “*About 1 to 1.5 hours”* were combined, while the other responses were maintained unaltered. Together they formed the categorical physical activity variable used in the analyses.

Change in physical activity from baseline to follow up was defined by a dichotomous variable – “Active/inactive” – created based on the physical activity variable as described above. Being active was defined as physical activity ≥2 h per week. Those who were active in both surveys were labelled “consistently active” and those who were inactive in both were labelled “consistently inactive”. The participants who became active between surveys (increased level of activity from < 2 h to ≥2 h per week) were labelled “adopters”. Participants who reduced their level of activity from ≥2 h to < 2 h per week were labelled “quitters”. A similar approach has been used in other studies [27, 28]. In addition to the primary exposures, we included baseline measurements of hours per weekday outside of school hours spent in front of a computer or TV (screen time), age in half years, study specialization (which was either general, sports or vocational subjects) and regularity of eating breakfast in the analyses as possible confounders.

Puberty is associated with body composition in adolescents, but in this particular cohort, data from the Pubertal Development Scale (PDS) was missing in a substantial number (17.8%) of boys. We explored the effect of adjusting for PDS or age at menarche (in girls) in complete case analyses, but as this had no substantial impact on results, we did not include the variables in the final model.

### Statistics

Results are presented sex-specific. We used descriptive statistics to determine the prevalence of overweight and obesity, levels of physical activity, mean values of BMI, waist circumference, FMI and LMI at baseline and follow-up as well as changes in BMI, waist circumference, FMI and LMI. Categorical variables were presented as proportions in percentages with number of subjects (n), while continuous variables were presented as means with standard deviation (SD) (Table 1). The associations between baseline physical activity and longitudinal changes in BMI, waist circumference, FMI and LMI were assessed using linear regression, with hours of physical activity coded to reflect the number of hours they represent. The associations with changes in activity status were assessed by analysis of covariance. As current body composition may affect the associations between physical activity and change in body composition, we adjusted all analyses for the baseline values. In the fully adjusted model we also included baseline measurements of sedentary behavior (screen time), study specialization, regularity of eating breakfast and age in half years, in addition to the time between baseline and follow-ups. We have presented adjusted beta coefficients for change in outcome at each level of physical activity at baseline (Table 2) or change in activity status (Table 3 and Table 4 in Appendix). A *p*-value of less than 0.05 was considered significant.

**Table 1:** Characteristics of the longitudinal cohort of the Tromsø Study; Fit Futures 2010–11 and Fit Futures 2012–13^a^

	Boys (*n* = 292)		Girls (*n* = 354)	
	FF1	FF2	FF1	FF2
Age (years)	16.1 (0.4)	18.2 (0.4)	16.1 (0.4)	18.2 (0.4)
Height (cm)	177.3 (6.5)	179.1 (6.5)	165.0 (6.5)	165.7 (6.6)
Body weight (kg)	69.9 (13.7)	75.3 (14.7)	60.4 (10.7)	63.1 (12.0)
Body mass index (BMI)	22.2 (3.9)	23.4 (4.2)	22.2 (3.8)	23.0 (4.2)
Body weight category^b^				
Underweight (BMI < 18.5)	8.6 (25)	8.2 (24)	5.9 (21)	4.5 (16)
Normal weight (18.5 ≤ BMI < 25)	70.2 (205)	63.7 (186)	75.1 (266)	74.6 (264)
Overweight (25 ≤ BMI < 30)	14.7 (43)	19.9 (58)	14.1 (50)	14.7 (52)
Obese (BMI ≥ 30)	6.5 (19)	8.2 (24)	4.8 (17)	6.2 (22)
Waist circumference (cm)	81.5 (11.0)	84.7 (11.8)	76.6 (9.6)	77.7 (11.1)
Total Body Fat Mass (kg)	14.3 (10.6)	16.7 (11.6)	19.8 (8.2)	21.6 (9.3)
Fat Mass Index (FMI)	4.5 (3.3)	5.2 (3.5)	7.3 (3.1)	7.9 (3.4)
Total Body Lean Mass (kg)	53.8 (6.6)	56.1 (7.0)	38.6 (4.5)	39.2 (4.8)
Lean Mass Index (LMI)	17.1 (1.6)	17.5 (1.8)	14.2 (1.3)	14.2 (1.4)
Regularity of eating breakfast				
Rarely/never	12.1 (35)	14.0 (39)	11.1 (39)	11.7 (41)
1–3 times weekly	14.8 (43)	15.8 (44)	15.0 (53)	17.1 (60)
4–6 times weekly	20.3 (59)	25.5 (71)	19.8 (70)	24.2 (85)
Daily	52.8 (153)	44.8 (125)	54.1 (191)	47.0 (165)
Screen time (hours per weekday)				
0–0.5 h	3.8 (11)	5.0 (14)	3.7 (13)	4.6 (16)
1–1.5 h	12.3 (36)	14.2 (40)	24.7 (87)	27.9 (98)
2–3 h	38.4 (112)	31.0 (87)	40.3 (142)	37.3 (131)
4-6 h	37.0 (108)	38.1 (107)	25.0 (88)	21.9 (77)
**≥** 7 h	8.6 (25)	11.7 (33)	6.3 (22)	8.3 (29)
Leisure time physical activity (hours per week)				
Inactive	30.5 (89)	37.1 (104)	27.4 (97)	36.2 (127)
0.5–1.5 h	8.9 (26)	8.2 (23)	8.5 (30)	12.0 (42)
2 to 3 h	16.8 (49)	11.4 (32)	22.6 (80)	16.5 (58)
4 to 6 h	23.6 (69)	21.8 (61)	27.1 (96)	23.1 (81)
≥ 7 h	20.2 (59)	21.4 (60)	14.4 (51)	12.3 (43)
Activity status: active^c^	60.6 (177)	54.6 (153)	64.1 (227)	51.9 (182)
Change in activity status				
Consistently inactive		27.1 (76)		25.1 (88)
Quitters		18.2 (51)		23.1 (81)
Adopters		11.8 (33)		11.1 (39)
Consistently active		42.9 (120)		40.7 (143)

^a^Values are means with standard deviation (SD) or prevalence in percentages (n). *BM*I Body weight in kg/height in meters^2^, *FMI* Fat mass in kg/height in meters^2^, *LMI* Lean mass in kg/height in meters^2^

^b^BMI (kg/m^2^) categories according to the International Obesity Task Force reference-standard [24, 25]

^c^Participants with 2 h or more of physical activity in leisure time per week

**Table 2:** Difference in BMI (kg/m^2^), waist circumference, FMI (fat mass in kg/m^2^) and LMI (lean mass in kg/m^2^) between Fit Futures 1 (2010–2011) and Fit Futures 2 (2012–2013), according to hours per week of physical activity in leisure time at baseline^a^

		Beta for ∆BMI (95% CI)				Beta for ∆waist circumference (95% CI)				Beta for ∆FMI (95% CI)				Beta for ∆LMI (95% CI)			
Boys	n	Model 1		Model 2		Model 1		Model 2		Model 1		Model 2		Model 1		Model 2	
Baseline physical activity	290	Beta	95% CI	Beta	95% CI	Beta	95% CI	Beta	95% CI	Beta	95% CI	Beta	95% CI	Beta	95% CI	Beta	95% CI
Inactive^b^	89	0		0		0		0		0		0		0		0	
About 0.5–1.5 h	26	0.30	− 0.54, 1.13	0.18	− 0.65, 1.01	0.61	−2.25, 3.47	0.49	−2.38, 3.35	0.04	− 0.74, 0.82	− 0.02	− 0.80, 0.76	0.30	− 0.05, 0.65	0.26	− 0.09, 0.61
About 2 to 3 h	48	0.12	− 0.56, 0.80	0.12	− 0.55, 0.80	− 0.11	− 2.43, 2.21	−0.11	− 2.44, 2.23	0.01	− 0.62, 0.64	0.01	− 0.63, 0.64	0.07	− 0.21, 0.35	0.09	− 0.20, 0.37
About 4 to 6 h	68	0.10	− 0.50, 0.71	− 0.09	− 0.70, 0.53	− 0.67	− 2.74, 1.40	− 0.84	− 2.96, 1.27	0.07	− 0.49, 0.64	− 0.06	− 0.64, 0.52	0.01	− 0.25, 0.27	− 0.05	− 0.31, 0.22
≥ 7 h	59	− 0.07	− 0.70, 0.57	− 0.48	− 1.24, 0.29	− 0.98	−3.16, 1.19	− 2.54	− 5.19, 0.12	−0.30	− 0.91, 0.30	− 0.52	− 1.25, 0.21	0.20	− 0.08, 0.48	0.05	− 0.28, 0.38
P for linear trend		0.77		0.20		0.25		0.05*		0.41		0.22		0.41		0.75	
Girls																	
Baseline physical activity	351																
Inactive^c^	95	0		0		0		0		0		0		0		0	
About 0.5–1.5 h	30	0.26	− 0.50, 1.01	0.40	− 0.37, 1.17	0.91	−2.07, 3.89	1.29	−1.76, 4.34	0.38	− 0.31, 1.06	0.56	− 0.13, 1.25	0.03	− 0.25, 0.30	− 0.01	− 0.29, 0.27
About 2 to 3 h	80	0.03	− 0.52, 0.57	0.09	− 0.47, 0.64	0.01	− 2.15, 2.17	0.14	− 2.06, 2.33	0.14	− 0.36, 0.63	0.21	− 0.28, 0.71	− 0.02	− 0.22, 0.18	− 0.03	− 0.23, 0.17
About 4 to 6 h	95	− 0.40	− 0.93, 0.12	− 0.25	− 0.82, 0.32	− 0.10	− 2.17, 1.97	0.23	−2.02, 2.49	− 0.30	− 0.78, 0.17	− 0.12	− 0.63, 0.39	− 0.05	− 0.24, 0.15	− 0.08	− 0.29, 0.13
≥ 7 h	51	0.51	− 0.12, 1.14	0.74	0.04, 1.44*	2.16	− 0.33, 4.64	2.80	0.02, 5.57*	0.60	0.03, 1.18*	0.90	0.27, 1.53*	0.01	− 0.22, 0.24	− 0.04	− 0.30, 0.22
P for linear trend		0.69		0.34		0.23		0.15		0.48		0.14		0.88		0.60	

*Significantly different from the reference (*p* < 0.05)

^a^Model 1 adjusted for baseline measurement of outcome. Model 2 adjusted for baseline measurement of outcome, screen time on weekdays, regularity of eating breakfast, age in half years at baseline and days between measurements

^b^Inactive boys had a mean increase of 1.1 BMI units, 3.2 cm waist circumference, 0.6 FMI units and 0.3 LMI units

^c^Inactive girls had a mean increase of 0.8 BMI units, 0.6 cm waist circumference, 0.5 FMI units and 0.1 LMI units

**Table 3:** Difference in BMI (kg/m^2^), waist circumference, FMI (fat mass in kg/m^2^) and LMI (lean mass in kg/m^2^) between Fit Futures 1 (2010–2011) and Fit Futures 2 (2012–2013) according to change in activity status between the surveys^a^

Boys	n	Beta for ∆BMI (95% CI)		Beta for ∆waist circumference (95% CI)		Beta for ∆FMI (95% CI)		Beta for ∆LMI (95% CI)	
Change in activity status	278	Model 1	Model 2	Model 1	Model 2	Model 1	Model 2	Model 1	Model 2
Consistently inactive^b^	76	0	0	0	0	0	0	0	0
Quitters	50	0.15 (− 0.53, 0.82)	0.06 (− 0.61, 0.73)	− 0.11 (−2.44, 2.21)	−0.29 (− 2.63, 2.04)	0.08 (− 0.54, 0.70)	0.02 (− 0.61, 0.64)	0.02 (− 0.27, 0.30)	− 0.01 (− 0.29, 0.28)
Adopters	33	− 0.54 (− 1.32, 0.24)	− 0.72 (− 1.49, 0.06)	− 2.00 (− 4.67, 0.66)	−2.39 (− 5.08, 0.30)	− 0.93 (− 1.64, − 0.22)*	− 1.04 (− 1.76, − 0.32)*	0.33 (− 0.00, 0.66)	0.29 (− 0.04, 0.62)
Consistently active	119	− 0.17 (− 0.71, 0.38)	− 0.47 (− 1.07, 0.13)	− 1.46 (− 3.33, 0.41)	− 2.32 (− 4.40, − 0.24)*	− 0.42 (− 0.92, 0.09)	− 0.62 (− 1.17, − 0.06)*	0.22 (− 0.03, 0.46)	0.13 (− 0.13, 0.40)
ANOVA F-test		0.40	0.13	0.26	0.08	0.03	< 0.01	0.11	0.29
Girls									
Change in activity status	348								
Consistently inactive^c^	86	0	0	0	0	0	0	0	0
Quitters	80	0.14 (− 0.43, 0.70)	0.14 (− 0.43, 0.72)	0.48 (−1.75, 2.70)	0.39 (− 1.88, 2.65)	0.30 (− 0.21, 0.81)	0.31 (− 0.21, 0.82)	− 0.10 (− 0.30, 0.11)	− 0.09 (− 0.30, 0.12)
Adopters	39	0.05 (− 0.66, 0.75)	0.05 (− 0.66, 0.77)	− 0.36 (− 3.14, 2.41)	− 0.52 (− 3.33, 2.30)	− 0.09 (− 0.73, 0.54)	− 0.09 (− 0.73, 0.55)	0.23 (− 0.02, 0.47)	0.23 (− 0.02, 0.49)
Consistently active	143	− 0.22 (− 0.72, 0.28)	− 0.14 (− 0.69, 0.41)	− 0.04 (− 2.01, 1.92)	− 0.02 (− 2.19, 2.16)	− 0.30 (− 0.75, 0.15)	− 0.22 (− 0.72, 0.27)	0.13 (− 0.05, 0.31)	0.13 (− 0.06, 0.33)
ANOVA F-test		0.54	0.76	0.94	0.94	0.09	0.19	0.02	0.04

*Significantly different from the reference (*p* < 0.05)

^a^Change in outcome in categories of activity status relative to consistently inactive as reference, and with an F-test for difference between groups. Model 1 adjusted for baseline measurement of outcome. Model 2 adjusted for baseline measurement of outcome, screen time on weekdays, regularity of eating breakfast, age in half years at baseline and days between measurements

^b^Consistently inactive boys had a mean increase of 1.3 BMI units, 3.9 cm waist circumference, 0.9 FMI units and 0.3 LMI units

^c^Consistently inactive girls had a mean increase of 0.8 BMI units, 0.9 cm waist circumference, 0.6 FMI units and 0.1 LMI units

All statistical analyses were performed using STATA, version 14 (StataCorp, College Station, Texas, USA).

## Results

Table 1 shows the descriptive characteristics of the study population. Mean BMI increased by 1.2 units for boys, and 0.8 units for girls between the surveys. On average, boys experienced a larger increase of both body height and body weight than girls. In boys, the combined prevalence of overweight and obesity (BMI ≥ 25) increased from 21.2 to 28.1%, while for girls it increased from 18.9 to 20.9%. Waist circumference increased less in girls (1.1 cm) than in boys (3.2 cm). Both sexes experienced a similar increase in FMI (0.7 kg/m^2^ in boys and 0.6 kg/m^2^ in girls). Boys experienced a small increase in LMI (0.4 kg/m^2^), whereas in girls there was no change. The proportion of adolescents classified as active in leisure time (active ≥ 2 h per week) decreased by 6%-points for boys and 12.2%-points for girls between the surveys.

There was no statistically significant linear effect of physical activity levels reported in 2010–2011 on change in neither BMI, FMI nor LMI during the following 2 years (Table 2). This was true for both sexes and also after adjustments. There were indications of a linear, inverse relationship with waist circumference in boys (*p* = 0.05), whereas a non-significant positive relationship was seen in girls. The most active boys gained less in BMI, waist circumference and FMI relative to the inactive, albeit not statistically significant. In contrast, the most active girls experienced a statistically significant higher adjusted increase in BMI (0.74 (95% CI: 0.04, 1.44)), waist circumference (2.80 (95% CI: 0.02, 5.57)) and FMI (0.90 (95% CI: 0.27, 1.53)) compared to the inactive girls. Stratified analyses including only girls who were active more than 6 h per week at baseline showed no difference in mean increase of BMI, FMI or waist circumference in consistently active girls compared to girls who reduced their level of physical activity. In boys, LMI increased most in those who at baseline were active between 0.5 and 1.5 h per week, but the increase was not significantly different from that observed among the inactive (0.26 (95% CI: − 0.09, 0.61)). In girls, change in LMI differed little across level of activity.

Table 3 presents changes in BMI, waist circumference, FMI and LMI according to change in activity status from 2010 to 2011 to 2012–2013. In both sexes, neither quitting activity nor adopting activity, relative to remaining inactive, was significantly associated with change in BMI or waist circumference. The consistently active boys had a significantly lower increase in waist circumference compared to the consistently inactive (− 2.32 (95% CI: − 4.40, − 0.24)). The largest increase in BMI and FMI (and for girls, also waist circumference) was observed among those quitting activity during follow-up, but this was not statistically significantly different from change among those who remained inactive.

In boys, changes in FMI were significantly different between activity groups (*p* < 0.01), with adopters (− 1.04 (95% CI -1.76, − 0.32)) and the consistently active (− 0.62 (95% CI: − 1.17, − 0.06)) gaining significantly less FMI than the consistently inactive. The difference in change in FMI comparing adopters and quitters was also statistically significant (− 1.06 (95% CI: − 1.83, − 0.28)) (Table 4 in Appendix). In girls there was no statistically significant difference in change of FMI between categories of activity, with the exception of the consistently active which gained less than those quitting activity (− 0.53 (95% CI: − 1.00, − 0.05)) (Table 4 in Appendix).

In boys, there was no statistically significant difference in change in LMI between the groups. In girls, change in LMI differed significantly between groups (*p* = 0.04). Girls who adopted activity between surveys experienced greater increase in LMI than the consistently inactive, but the difference was not of statistical significance (0.23 (95% CI: − 0.02, 0.49)). Compared to those quitting activity, girls who were consistently active (0.22 (95% CI: 0.03, 0.41)) or adopted physical activity (0.32 (95% CI: 0.07, 0.58)) experienced a statistically significantly higher increase in LMI (Table 4 in Appendix).

## Discussion

In this population-based longitudinal study of changes in body composition in adolescents, there was, with the exception of waist circumference in boys, no linear association between self-reported leisure time physical activity and 2-year changes in indices of body composition. Change in physical activity was associated with statistically significant different changes in FMI. Boys who increased their physical activity during follow-up decreased their FMI compared to groups of boys quitting or remaining inactive, while consistently active girls experienced less increase than those reducing activity. Change in physical activity in girls was associated with statistically significant different changes in LMI. Girls who adopted physical activity increased their LMI compared to girls quitting activity.

Body weight, BMI and waist circumference increase during natural growth in children and adolescents, and it is therefore challenging to separate healthy- from unhealthy body development. Although the direction and magnitude of change will vary between individuals, a general increase in all the included measures of body composition is expected during this phase of life given the bodily- and hormonal changes that naturally takes place in adolescents [21]. Physical activity has positive health effects, but the associations with changes in adiposity among adolescents is complicated and conflicting results have been reported [29]. We found weak relationships between the frequency of leisure time physical activity at baseline and change in body composition, suggesting that change in body composition in this age group was mainly independent of level of self-reported physical activity. Girls who were most active at baseline had put on adipose tissue after 2 years (Table 2). A possible explanation could be that the increase occurred in girls who were active at baseline, but reduced their activity during follow up. Stratified analyses in categories of girls who were active more than 6 h per week at baseline did not support this explanation. Our findings are, however, in line with those of Kettaneh et al., who found that girls in the highest category of activity also experienced the largest increase in BMI, waist circumference, sum of skinfolds and percent body fat [17]. LMI remained unaltered between Fit Futures 1 and Fit Futures 2 (Table 1), suggesting that LMI changes little in females during late adolescence.

Lean mass is comprised of muscles and all bodily tissue except fat mass and skeletal mass. Since muscles are particularly important for oxidization of fat, they are also determinants of energy balance [30], and although physical activity increases muscle mass it is not the sole component of energy expenditure. Total energy expenditure is comprised of resting metabolic rate, the thermic effect of food, bodily movement and, for children and adolescents; energy required for growth [31]. This means that although physical activity declines, the effect on total energy expenditure is modest [17]. Adiposity is the result of a whole range of lifestyle-, sociocultural- and genetic factors. It is therefore difficult to pinpoint the impact of one behavior, and it is possible that factors other than physical activity – and changes in these, exert more influence on change in body composition [32].

Physical activity levels change rapidly in adolescents [33], thus challenging our ability to measure and capture the effect of physical activity on body composition in adolescents. Thus, a baseline measurement may be only modestly associated with prior- or future physical activity [31]. For instance, O’Loughlin et al. reported effects of physical activity on changes in adiposity after 1 year, but not 2 years in girls, and only after 2 years in boys. The authors hypothesized that change in levels of physical activity over follow-up may have contributed to the differences [34].

Boys adopting activity experienced a slight decrease in FMI between surveys. This finding differs from the observed increase in all other measures of body composition in both sexes, and in all other sub-groups of activity change. With the exception of waist circumference and FMI in boys, change in all measures of body composition among the consistently active did not differ statistically significantly from changes in the consistently inactive. Physical activity has a limited potential to affect the difference between these groups [32]. In the consistently inactive, there is less room for unhealthy weight gain as a result of inactivity. Conversely, among the consistently active there is less potential for preventing unhealthy weight gain through increased activity. These groups may be more susceptible to unhealthy weight gain through factors other than, or in addition to, physical activity. This can be considered as floor- and ceiling effects of physical activity, and means that the potential for activity related changes in adiposity is greatest among those who change their level of activity. The prevalence of physically active adolescents declined in our study, and for both sexes there was a rather consistent, albeit not statistically significant, pattern of the highest increase in BMI, waist circumference (not in boys) and FMI in those quitting activity. These findings indicate that those who reduce their level of activity over the course of adolescence are susceptible to unhealthy weight gain. This is of concern, since total activity decreases by 7% annually in adolescents [33]. Boys who adopted physical activity reduced their FMI between surveys and had the highest increase in LMI, indicating that the inactive may profit from increasing level of physical activity. In girls, we observed a statistically significant difference in change of FMI between those who were consistently active and those quitting activity, suggesting that there are negative consequences of reducing level of physical activity. However, girls naturally increase fat mass over the course of adolescence, whereas the same is true for lean mass in boys [17]. It is therefore possible that an increase in FMI in girls occurs regardless of activity level, whereas for boys, this may be prevented through activity. This can also explain why there was no significant associations between change in activity and change in BMI, as BMI does not distinguish between the overweight inactive (with high FMI) and the overweight active (with high LMI) [35].

Individuals may have, and report, high levels of physical activity because they try to lose weight, or they may have low (or high) body weight because of high activity. The problem of reverse causality applies also to longitudinal studies, as overweight adolescents may avoid engaging in physical activity on account of feeling inferior relative to their active peers [31, 36]. Self-reported physical activity is prone to information bias [26] and individuals tend to overestimate the true amount of their physical activity. This can potentially dilute an association with measures of body composition [15]. Furthermore, self-reported physical activity in leisure time does not capture the total level of activity, which can include active transportation to school and friends, physical education and other types of leisure time activity. Objective measures of physical activity can produce more accurate estimates, but are not necessarily associated with changes in adiposity [37]. Finally, studies have suggested that the intensity of activity is more important than the total amount of activity for adiposity [38, 39]. In our study, complete data on perceived physical activity intensity were not available, but in complete case analyses the inclusion of self-reported intensity did not affect results.

This study had several strengths, including the longitudinal design, the high participation rate and the inclusion of four objective measures of body composition. A limitation is the use of self-reported physical activity and the lack of full adjustment for dietary habits, since a validated food-frequency questionnaires or similar was not included in the study. Another limitation is the lack of adjustment for pubertal development due to missing data. However, in boys, the vast majority (≈73%) of complete cases reported pubertal maturation to be “underway”, meaning that the effect of adjusting for PDS would likely be small. Inclusion of PDS in complete case analyses did not indicate confounding by pubertal development. Another limitation is lack of adjustment for socioeconomic status. In the Fit Futures survey, a substantial number of participants reported not knowing parental level of education, thus limiting the possibilities for adjusting for this variable. However, the inclusion of study specialization in the analyses likely adjusts for some of the variance in socioeconomic status in adolescents [40, 41]. Lastly, in our study the length of follow-up was approximately 2 years, but in a population undergoing natural changes in body composition, it may take more time before physical inactivity manifests in body composition. The 3rd survey of the Fit Futures Study is in planning and will enable further research on how physical activity in late adolescence affects changes in body composition in early adulthood.

## Conclusion

In this longitudinal study of changes in objectively measured body composition, we found that consistently inactive boys increased significantly more in fat mass index compared to those adopting physical activity or remaining consistently active, and that girls adopting physical activity increased their lean mass index significantly more than those who reduced physical activity. Adolescence is a time of transformation and it is challenging to pinpoint the effect of one behavior on change in body composition. Physical activity should nevertheless be encouraged because of the health benefits other than the prevention of adiposity.

## Data Availability

The data that support the findings of this study are available from UiT – The Arctic University of Norway, but restrictions apply to the availability of these data, which were used under license for the current study, and so are not publicly available. Data are, however, available from the authors upon reasonable request and with permission of UiT – The Arctic University of Norway.

## Appendix

**Table 4:** Difference in BMI (kg/m^2^), waist circumference, FMI (fat mass in kg/m^2^) and LMI (lean mass in kg/m^2^) between Fit Futures 1 (2010–2011) and Fit Futures 2 (2012–2013) according to change in activity status between the surveys^a^

Boys	n	Beta for ∆BMI (95% CI)		Beta for ∆waist circumference (95% CI)		Beta for ∆FMI (95% CI)		Beta for ∆LMI (95% CI)	
Change in activity status	278	Model 1	Model 2	Model 1	Model 2	Model 1	Model 2	Model 1	Model 2
Quitters^b^	50	0	0	0	0	0	0	0	0
Consistently inactive	76	− 0.15 (−0.82, 0.53)	− 0.06 (− 0.73, 0.61)	0.11 (− 2.21, 2.44)	0.29 (− 2.04, 2.63)	− 0.08 (− 0.70, 0.54)	−0.02 (− 0.64, 0.61)	− 0.02 (− 0.30, 0.27)	0.01 (− 0.28, 0.29)
Adopters	33	− 0.69 (− 1.53, 0.15)	−0.78 (− 1.61,0 0.05)	−1.89 (− 4.79, 1.00)	−2.10 (− 5.00, 0.81)	− 1.01 (− 1.78, − 0.23)*	−1.06 (− 1.83, − 0.28)*	0.32 (− 0.04, 0.67)	0.29 (− 0.06, 0.65)
Consistently active	119	−0.31 (− 0.94, 0.31)	−0.54 (− 1.19, 0.11)	−1.35 (− 3.49, 0.80)	− 2.02 (− 4.28, 0.24)	− 0.50 (− 1.07, 0.08)	−0.63 (− 1.24, − 0.03)*	0.20 (− 0.07, 0.47)	0.14 (− 0.14, 0.42)
Girls									
Change in activity status	348								
Quitters^c^	80	0	0	0	0	0	0	0	0
Consistently inactive	86	− 0.14 (−0.70, 0.43)	− 0.14 (− 0.72, 0.43)	− 0.48 (−2.70, 1.75)	− 0.39 (− 2.65, 1.88)	− 0.30 (− 0.81, 0.21)	−0.31 (− 0.82, 0.21)	0.10 (− 0.11, 0.30)	0.09 (− 0.12, 0.30)
Adopters	39	− 0.09 (− 0.80, 0.63)	− 0.09 (− 0.80, 0.63)	− 0.84 (− 3.64, 1.96)	− 0.90 (− 3.71, 1.90)	− 0.39 (−1.04, 0.25)	−0.40 (− 1.04, 0.24)	0.32 (0.07, 0.58)*	0.32 (0.07, 0.58)*
Consistently active	143	− 0.36 (− 0.87, 0.16)	− 0.29 (− 0.82, 0.24)	− 0.52 (− 2.53, 1.49)	− 0.41 (− 2.49, 1.68)	− 0.60 (− 1.06, − 0.14)*	−0.53 (− 1.00, − 0.05)*	0.23 (0.05, 0.41)*	0.22 (0.03, 0.41)*

*Significantly different from the reference (*p* < 0.05)

^a^Change in outcome in categories of activity status relative to quitting activity as reference. Model 1 adjusted for baseline measurement of outcome. Model 2 adjusted for baseline measurement of outcome, screen time on weekdays, regularity of eating breakfast, age in half years at baseline and days between measurements

^b^Boys quitting activity had a mean increase of 1.5 BMI units, 4.1 cm waist circumference, 1.0 FMI units and 0.3 LMI units

^c^Girls quitting activity had a mean increase of 1.0 BMI units, 1.4 cm waist circumference, 0.9 FMI units and − 0.1 LMI units

## Abbreviations

BMI: Body Mass Index
FMI: Fat Mass Index
LMI: Lean Mass Index
PDS: Pubertal Development Scale

## References

[1] Engeland A, Bjørge T, Tverdal A, Søgaard AJ (2004). Obesity in adolescence and adulthood and the risk of adult mortality. Epidemiology..

[2] World Health Organization. Obesity and Overweight Fact Sheet. 2018. http://www.who.int/news-room/fact-sheets/detail/obesity-and-overweight. Accessed Dec 2018.

[3] Evensen Elin, Emaus Nina, Kokkvoll Ane, Wilsgaard Tom, Furberg Anne-Sofie, Skeie Guri (2017). The relation between birthweight, childhood body mass index, and overweight and obesity in late adolescence: a longitudinal cohort study from Norway, The Tromsø Study, Fit Futures. BMJ Open.

[4] Abarca-Gómez Leandra, Abdeen Ziad A, Hamid Zargar Abdul, Abu-Rmeileh Niveen M, Acosta-Cazares Benjamin, Acuin Cecilia, Adams Robert J, Aekplakorn Wichai, Afsana Kaosar, Aguilar-Salinas Carlos A, Agyemang Charles, Ahmadvand Alireza, Ahrens Wolfgang, Ajlouni Kamel, Akhtaeva Nazgul, Al-Hazzaa Hazzaa M, Al-Othman Amani Rashed, Al-Raddadi Rajaa, Al Buhairan Fadia, Al Dhukair Shahla, Ali Mohamed M, Ali Osman, Alkerwi Ala'a, Alvarez-Pedrerol Mar, Aly Eman, Amarapurkar Deepak N, Amouyel Philippe, Amuzu Antoinette, Andersen Lars Bo, Anderssen Sigmund A, Andrade Dolores S, Ängquist Lars H, Anjana Ranjit Mohan, Aounallah-Skhiri Hajer, Araújo Joana, Ariansen Inger, Aris Tahir, Arlappa Nimmathota, Arveiler Dominique, Aryal Krishna K, Aspelund Thor, Assah Felix K, Assunção Maria Cecília F, Aung May Soe, Avdicová Mária, Azevedo Ana, Azizi Fereidoun, Babu Bontha V, Bahijri Suhad, Baker Jennifer L, Balakrishna Nagalla, Bamoshmoosh Mohamed, Banach Maciej, Bandosz Piotr, Banegas José R, Barbagallo Carlo M, Barceló Alberto, Barkat Amina, Barros Aluisio JD, Barros Mauro VG, Bata Iqbal, Batieha Anwar M, Batista Rosangela L, Batyrbek Assembekov, Baur Louise A, Beaglehole Robert, Romdhane Habiba Ben, Benedics Judith, Benet Mikhail, Bennett James E, Bernabe-Ortiz Antonio, Bernotiene Gailute, Bettiol Heloisa, Bhagyalaxmi Aroor, Bharadwaj Sumit, Bhargava Santosh K, Bhatti Zaid, Bhutta Zulfiqar A, Bi Hongsheng, Bi Yufang, Biehl Anna, Bikbov Mukharram, Bista Bihungum, Bjelica Dusko J, Bjerregaard Peter, Bjertness Espen, Bjertness Marius B, Björkelund Cecilia, Blokstra Anneke, Bo Simona, Bobak Martin, Boddy Lynne M, Boehm Bernhard O, Boeing Heiner, Boggia Jose G, Boissonnet Carlos P, Bonaccio Marialaura, Bongard Vanina, Bovet Pascal, Braeckevelt Lien, Braeckman Lutgart, Bragt Marjolijn CE, Brajkovich Imperia, Branca Francesco, Breckenkamp Juergen, Breda João, Brenner Hermann, Brewster Lizzy M, Brian Garry R, Brinduse Lacramioara, Bruno Graziella, Bueno-de-Mesquita H B(as), Bugge Anna, Buoncristiano Marta, Burazeri Genc, Burns Con, de León Antonio Cabrera, Cacciottolo Joseph, Cai Hui, Cama Tilema, Cameron Christine, Camolas José, Can Günay, Cândido Ana Paula C, Capanzana Mario, Capuano Vincenzo, Cardoso Viviane C, Carlsson Axel C, Carvalho Maria J, Casanueva Felipe F, Casas Juan-Pablo, Caserta Carmelo A, Chamukuttan Snehalatha, Chan Angelique W, Chan Queenie, Chaturvedi Himanshu K, Chaturvedi Nishi, Chen Chien-Jen, Chen Fangfang, Chen Huashuai, Chen Shuohua, Chen Zhengming, Cheng Ching-Yu, Chetrit Angela, Chikova-Iscener Ekaterina, Chiolero Arnaud, Chiou Shu-Ti, Chirita-Emandi Adela, Chirlaque María-Dolores, Cho Belong, Cho Yumi, Christensen Kaare, Christofaro Diego G, Chudek Jerzy, Cifkova Renata, Cinteza Eliza, Claessens Frank, Clays Els, Concin Hans, Confortin Susana C, Cooper Cyrus, Cooper Rachel, Coppinger Tara C, Costanzo Simona, Cottel Dominique, Cowell Chris, Craig Cora L, Crujeiras Ana B, Cucu Alexandra, D'Arrigo Graziella, d'Orsi Eleonora, Dallongeville Jean, Damasceno Albertino, Damsgaard Camilla T, Danaei Goodarz, Dankner Rachel, Dantoft Thomas M, Dastgiri Saeed, Dauchet Luc, Davletov Kairat, De Backer Guy, De Bacquer Dirk, De Curtis Amalia, de Gaetano Giovanni, De Henauw Stefaan, de Oliveira Paula Duarte, De Ridder Karin, De Smedt Delphine, Deepa Mohan, Deev Alexander D, Dehghan Abbas, Delisle Hélène, Delpeuch Francis, Deschamps Valérie, Dhana Klodian, Di Castelnuovo Augusto F, Dias-da-Costa Juvenal Soares, Diaz Alejandro, Dika Zivka, Djalalinia Shirin, Do Ha TP, Dobson Annette J, Donati Maria Benedetta, Donfrancesco Chiara, Donoso Silvana P, Döring Angela, Dorobantu Maria, Dorosty Ahmad Reza, Doua Kouamelan, Drygas Wojciech, Duan Jia Li, Duante Charmaine, Duleva Vesselka, Dulskiene Virginija, Dzerve Vilnis, Dziankowska-Zaborszczyk Elzbieta, Egbagbe Eruke E, Eggertsen Robert, Eiben Gabriele, Ekelund Ulf, El Ati Jalila, Elliott Paul, Engle-Stone Reina, Erasmus Rajiv T, Erem Cihangir, Eriksen Louise, Eriksson Johan G, la Peña Jorge Escobedo-de, Evans Alun, Faeh David, Fall Caroline H, Sant'Angelo Victoria Farrugia, Farzadfar Farshad, Felix-Redondo Francisco J, Ferguson Trevor S, Fernandes Romulo A, Fernández-Bergés Daniel, Ferrante Daniel, Ferrari Marika, Ferreccio Catterina, Ferrieres Jean, Finn Joseph D, Fischer Krista, Flores Eric Monterubio, Föger Bernhard, Foo Leng Huat, Forslund Ann-Sofie, Forsner Maria, Fouad Heba M, Francis Damian K, Franco Maria do Carmo, Franco Oscar H, Frontera Guillermo, Fuchs Flavio D, Fuchs Sandra C, Fujita Yuki, Furusawa Takuro, Gaciong Zbigniew, Gafencu Mihai, Galeone Daniela, Galvano Fabio, Garcia-de-la-Hera Manoli, Gareta Dickman, Garnett Sarah P, Gaspoz Jean-Michel, Gasull Magda, Gates Louise, Geiger Harald, Geleijnse Johanna M, Ghasemian Anoosheh, Giampaoli Simona, Gianfagna Francesco, Gill Tiffany K, Giovannelli Jonathan, Giwercman Aleksander, Godos Justyna, Gogen Sibel, Goldsmith Rebecca A, Goltzman David, Gonçalves Helen, González-Leon Margot, González-Rivas Juan P, Gonzalez-Gross Marcela, Gottrand Frederic, Graça Antonio Pedro, Graff-Iversen Sidsel, Grafnetter Dušan, Grajda Aneta, Grammatikopoulou Maria G, Gregor Ronald D, Grodzicki Tomasz, Grøntved Anders, Grosso Giuseppe, Gruden Gabriella, Grujic Vera, Gu Dongfeng, Gualdi-Russo Emanuela, Guallar-Castillón Pilar, Guan Ong Peng, Gudmundsson Elias F, Gudnason Vilmundur, Guerrero Ramiro, Guessous Idris, Guimaraes Andre L, Gulliford Martin C, Gunnlaugsdottir Johanna, Gunter Marc, Guo Xiuhua, Guo Yin, Gupta Prakash C, Gupta Rajeev, Gureje Oye, Gurzkowska Beata, Gutierrez Laura, Gutzwiller Felix, Hadaegh Farzad, Hadjigeorgiou Charalambos A, Si-Ramlee Khairil, Halkjær Jytte, Hambleton Ian R, Hardy Rebecca, Kumar Rachakulla Hari, Hassapidou Maria, Hata Jun, Hayes Alison J, He Jiang, Heidinger-Felso Regina, Heinen Mirjam, Hendriks Marleen Elisabeth, Henriques Ana, Cadena Leticia Hernandez, Herrala Sauli, Herrera Victor M, Herter-Aeberli Isabelle, Heshmat Ramin, Hihtaniemi Ilpo Tapani, Ho Sai Yin, Ho Suzanne C, Hobbs Michael, Hofman Albert, Hopman Wilma M, Horimoto Andrea RVR, Hormiga Claudia M, Horta Bernardo L, Houti Leila, Howitt Christina, Htay Thein Thein, Htet Aung Soe, Htike Maung Maung Than, Hu Yonghua, Huerta José María, Petrescu Constanta Huidumac, Huisman Martijn, Husseini Abdullatif, Huu Chinh Nguyen, Huybrechts Inge, Hwalla Nahla, Hyska Jolanda, Iacoviello Licia, Iannone Anna G, Ibarluzea Jesús M, Ibrahim Mohsen M, Ikeda Nayu, Ikram M Arfan, Irazola Vilma E, Islam Muhammad, Ismail Aziz al-Safi, Ivkovic Vanja, Iwasaki Masanori, Jackson Rod T, Jacobs Jeremy M, Jaddou Hashem, Jafar Tazeen, Jamil Kazi M, Jamrozik Konrad, Janszky Imre, Jarani Juel, Jasienska Grazyna, Jelakovic Ana, Jelakovic Bojan, Jennings Garry, Jeong Seung-Lyeal, Jiang Chao Qiang, Jiménez-Acosta Santa Magaly, Joffres Michel, Johansson Mattias, Jonas Jost B, Jørgensen Torben, Joshi Pradeep, Jovic Dragana P, Józwiak Jacek, Juolevi Anne, Jurak Gregor, Jureša Vesna, Kaaks Rudolf, Kafatos Anthony, Kajantie Eero O, Kalter-Leibovici Ofra, Kamaruddin Nor Azmi, Kapantais Efthymios, Karki Khem B, Kasaeian Amir, Katz Joanne, Kauhanen Jussi, Kaur Prabhdeep, Kavousi Maryam, Kazakbaeva Gyulli, Keil Ulrich, Boker Lital Keinan, Keinänen-Kiukaanniemi Sirkka, Kelishadi Roya, Kelleher Cecily, Kemper Han CG, Kengne Andre P, Kerimkulova Alina, Kersting Mathilde, Key Timothy, Khader Yousef Saleh, Khalili Davood, Khang Young-Ho, Khateeb Mohammad, Khaw Kay-Tee, Khouw Ilse MSL, Kiechl-Kohlendorfer Ursula, Kiechl Stefan, Killewo Japhet, Kim Jeongseon, Kim Yeon-Yong, Klimont Jeannette, Klumbiene Jurate, Knoflach Michael, Koirala Bhawesh, Kolle Elin, Kolsteren Patrick, Korrovits Paul, Kos Jelena, Koskinen Seppo, Kouda Katsuyasu, Kovacs Viktoria A, Kowlessur Sudhir, Koziel Slawomir, Kratzer Wolfgang, Kriemler Susi, Kristensen Peter Lund, Krokstad Steinar, Kromhout Daan, Kruger Herculina S, Kubinova Ruzena, Kuciene Renata, Kuh Diana, Kujala Urho M, Kulaga Zbigniew, Kumar R Krishna, Kunešová Marie, Kurjata Pawel, Kusuma Yadlapalli S, Kuulasmaa Kari, Kyobutungi Catherine, La Quang Ngoc, Laamiri Fatima Zahra, Laatikainen Tiina, Lachat Carl, Laid Youcef, Lam Tai Hing, Landrove Orlando, Lanska Vera, Lappas Georg, Larijani Bagher, Laugsand Lars E, Lauria Laura, Laxmaiah Avula, Bao Khanh Le Nguyen, Le Tuyen D, Lebanan May Antonnette O, Leclercq Catherine, Lee Jeannette, Lee Jeonghee, Lehtimäki Terho, León-Muñoz Luz M, Levitt Naomi S, Li Yanping, Lilly Christa L, Lim Wei-Yen, Lima-Costa M Fernanda, Lin Hsien-Ho, Lin Xu, Lind Lars, Linneberg Allan, Lissner Lauren, Litwin Mieczyslaw, Liu Jing, Loit Helle-Mai, Lopes Luis, Lorbeer Roberto, Lotufo Paulo A, Lozano José Eugenio, Luksiene Dalia, Lundqvist Annamari, Lunet Nuno, Lytsy Per, Ma Guansheng, Ma Jun, Machado-Coelho George LL, Machado-Rodrigues Aristides M, Machi Suka, Maggi Stefania, Magliano Dianna J, Magriplis Emmanuella, Mahaletchumy Alagappan, Maire Bernard, Majer Marjeta, Makdisse Marcia, Malekzadeh Reza, Malhotra Rahul, Rao Kodavanti Mallikharjuna, Malyutina Sofia, Manios Yannis, Mann Jim I, Manzato Enzo, Margozzini Paula, Markaki Anastasia, Markey Oonagh, Marques Larissa P, Marques-Vidal Pedro, Marrugat Jaume, Martin-Prevel Yves, Martin Rosemarie, Martorell Reynaldo, Martos Eva, Marventano Stefano, Masoodi Shariq R, Mathiesen Ellisiv B, Matijasevich Alicia, Matsha Tandi E, Mazur Artur, Mbanya Jean Claude N, McFarlane Shelly R, McGarvey Stephen T, McKee Martin, McLachlan Stela, McLean Rachael M, McLean Scott B, McNulty Breige A, Yusof Safiah Md, Mediene-Benchekor Sounnia, Medzioniene Jurate, Meirhaeghe Aline, Meisfjord Jørgen, Meisinger Christa, Menezes Ana Maria B, Menon Geetha R, Mensink Gert BM, Meshram Indrapal I, Metspalu Andres, Meyer Haakon E, Mi Jie, Michaelsen Kim F, Michels Nathalie, Mikkel Kairit, Miller Jody C, Minderico Cláudia S, Miquel Juan Francisco, Miranda J Jaime, Mirkopoulou Daphne, Mirrakhimov Erkin, Mišigoj-Durakovic Marjeta, Mistretta Antonio, Mocanu Veronica, Modesti Pietro A, Mohamed Mostafa K, Mohammad Kazem, Mohammadifard Noushin, Mohan Viswanathan, Mohanna Salim, Yusoff Muhammad Fadhli Mohd, Molbo Drude, Møllehave Line T, Møller Niels C, Molnár Dénes, Momenan Amirabbas, Mondo Charles K, Monterrubio Eric A, Monyeki Kotsedi Daniel K, Moon Jin Soo, Moreira Leila B, Morejon Alain, Moreno Luis A, Morgan Karen, Mortensen Erik Lykke, Moschonis George, Mossakowska Malgorzata, Mostafa Aya, Mota Jorge, Mota-Pinto Anabela, Motlagh Mohammad Esmaeel, Motta Jorge, Mu Thet Thet, Muc Magdalena, Muiesan Maria Lorenza, Müller-Nurasyid Martina, Murphy Neil, Mursu Jaakko, Murtagh Elaine M, Musil Vera, Nabipour Iraj, Nagel Gabriele, Naidu Balkish M, Nakamura Harunobu, Námešná Jana, Nang Ei Ei K, Nangia Vinay B, Nankap Martin, Narake Sameer, Nardone Paola, Navarrete-Muñoz Eva Maria, Neal William A, Nenko Ilona, Neovius Martin, Nervi Flavio, Nguyen Chung T, Nguyen Nguyen D, Nguyen Quang Ngoc, Nieto-Martínez Ramfis E, Ning Guang, Ninomiya Toshiharu, Nishtar Sania, Noale Marianna, Noboa Oscar A, Norat Teresa, Norie Sawada, Noto Davide, Nsour Mohannad Al, O'Reilly Dermot, Obreja Galina, Oda Eiji, Oehlers Glenn, Oh Kyungwon, Ohara Kumiko, Olafsson Örn, Olinto Maria Teresa Anselmo, Oliveira Isabel O, Oltarzewski Maciej, Omar Mohd Azahadi, Onat Altan, Ong Sok King, Ono Lariane M, Ordunez Pedro, Ornelas Rui, Ortiz Ana P, Osler Merete, Osmond Clive, Ostojic Sergej M, Ostovar Afshin, Otero Johanna A, Overvad Kim, Owusu-Dabo Ellis, Paccaud Fred Michel, Padez Cristina, Pahomova Elena, Pajak Andrzej, Palli Domenico, Palloni Alberto, Palmieri Luigi, Pan Wen-Harn, Panda-Jonas Songhomitra, Pandey Arvind, Panza Francesco, Papandreou Dimitrios, Park Soon-Woo, Parnell Winsome R, Parsaeian Mahboubeh, Pascanu Ionela M, Patel Nikhil D, Pecin Ivan, Pednekar Mangesh S, Peer Nasheeta, Peeters Petra H, Peixoto Sergio Viana, Peltonen Markku, Pereira Alexandre C, Perez-Farinos Napoleon, Pérez Cynthia M, Peters Annette, Petkeviciene Janina, Petrauskiene Ausra, Peykari Niloofar, Pham Son Thai, Pierannunzio Daniela, Pigeot Iris, Pikhart Hynek, Pilav Aida, Pilotto Lorenza, Pistelli Francesco, Pitakaka Freda, Piwonska Aleksandra, Plans-Rubió Pedro, Poh Bee Koon, Pohlabeln Hermann, Pop Raluca M, Popovic Stevo R, Porta Miquel, Portegies Marileen LP, Posch Georg, Poulimeneas Dimitrios, Pouraram Hamed, Pourshams Akram, Poustchi Hossein, Pradeepa Rajendra, Prashant Mathur, Price Jacqueline F, Puder Jardena J, Pudule Iveta, Puiu Maria, Punab Margus, Qasrawi Radwan F, Qorbani Mostafa, Bao Tran Quoc, Radic Ivana, Radisauskas Ricardas, Rahman Mahfuzar, Rahman Mahmudur, Raitakari Olli, Raj Manu, Rao Sudha Ramachandra, Ramachandran Ambady, Ramke Jacqueline, Ramos Elisabete, Ramos Rafel, Rampal Lekhraj, Rampal Sanjay, Rascon-Pacheco Ramon A, Redon Josep, Reganit Paul Ferdinand M, Ribas-Barba Lourdes, Ribeiro Robespierre, Riboli Elio, Rigo Fernando, de Wit Tobias F Rinke, Rito Ana, Ritti-Dias Raphael M, Rivera Juan A, Robinson Sian M, Robitaille Cynthia, Rodrigues Daniela, Rodríguez-Artalejo Fernando, del Cristo Rodriguez-Perez María, Rodríguez-Villamizar Laura A, Rojas-Martinez Rosalba, Rojroongwasinkul Nipa, Romaguera Dora, Ronkainen Kimmo, Rosengren Annika, Rouse Ian, Roy Joel GR, Rubinstein Adolfo, Rühli Frank J, Ruiz-Betancourt Blanca Sandra, Russo Paola, Rutkowski Marcin, Sabanayagam Charumathi, Sachdev Harshpal S, Saidi Olfa, Salanave Benoit, Martinez Eduardo Salazar, Salmerón Diego, Salomaa Veikko, Salonen Jukka T, Salvetti Massimo, Sánchez-Abanto Jose, Sandjaja, Sans Susana, Marina Loreto Santa, Santos Diana A, Santos Ina S, Santos Osvaldo, dos Santos Renata Nunes, Santos Rute, Saramies Jouko L, Sardinha Luis B, Sarrafzadegan Nizal, Saum Kai-Uwe, Savva Savvas, Savy Mathilde, Scazufca Marcia, Rosario Angelika Schaffrath, Schargrodsky Herman, Schienkiewitz Anja, Schipf Sabine, Schmidt Carsten O, Schmidt Ida Maria, Schultsz Constance, Schutte Aletta E, Sein Aye Aye, Sen Abhijit, Senbanjo Idowu O, Sepanlou Sadaf G, Serra-Majem Luis, Shalnova Svetlana A, Sharma Sanjib K, Shaw Jonathan E, Shibuya Kenji, Shin Dong Wook, Shin Youchan, Shiri Rahman, Siani Alfonso, Siantar Rosalynn, Sibai Abla M, Silva Antonio M, Silva Diego Augusto Santos, Simon Mary, Simons Judith, Simons Leon A, Sjöberg Agneta, Sjöström Michael, Skovbjerg Sine, Slowikowska-Hilczer Jolanta, Slusarczyk Przemyslaw, Smeeth Liam, Smith Margaret C, Snijder Marieke B, So Hung-Kwan, Sobngwi Eugène, Söderberg Stefan, Soekatri Moesijanti YE, Solfrizzi Vincenzo, Sonestedt Emily, Song Yi, Sørensen Thorkild IA, Soric Maroje, Jérome Charles Sossa, Soumare Aicha, Spinelli Angela, Spiroski Igor, Staessen Jan A, Stamm Hanspeter, Starc Gregor, Stathopoulou Maria G, Staub Kaspar, Stavreski Bill, Steene-Johannessen Jostein, Stehle Peter, Stein Aryeh D, Stergiou George S, Stessman Jochanan, Stieber Jutta, Stöckl Doris, Stocks Tanja, Stokwiszewski Jakub, Stratton Gareth, Stronks Karien, Strufaldi Maria Wany, Suárez-Medina Ramón, Sun Chien-An, Sundström Johan, Sung Yn-Tz, Sunyer Jordi, Suriyawongpaisal Paibul, Swinburn Boyd A, Sy Rody G, Szponar Lucjan, Tai E Shyong, Tammesoo Mari-Liis, Tamosiunas Abdonas, Tan Eng Joo, Tang Xun, Tanser Frank, Tao Yong, Tarawneh Mohammed Rasoul, Tarp Jakob, Tarqui-Mamani Carolina B, Tautu Oana-Florentina, Braunerová Radka Taxová, Taylor Anne, Tchibindat Félicité, Theobald Holger, Theodoridis Xenophon, Thijs Lutgarde, Thuesen Betina H, Tjonneland Anne, Tolonen Hanna K, Tolstrup Janne S, Topbas Murat, Topór-Madry Roman, Tormo María José, Tornaritis Michael J, Torrent Maties, Toselli Stefania, Traissac Pierre, Trichopoulos Dimitrios, Trichopoulou Antonia, Trinh Oanh TH, Trivedi Atul, Tshepo Lechaba, Tsigga Maria, Tsugane Shoichiro, Tulloch-Reid Marshall K, Tullu Fikru, Tuomainen Tomi-Pekka, Tuomilehto Jaakko, Turley Maria L, Tynelius Per, Tzotzas Themistoklis, Tzourio Christophe, Ueda Peter, Ugel Eunice E, Ukoli Flora AM, Ulmer Hanno, Unal Belgin, Uusitalo Hannu MT, Valdivia Gonzalo, Vale Susana, Valvi Damaskini, van der Schouw Yvonne T, Van Herck Koen, Van Minh Hoang, van Rossem Lenie, Van Schoor Natasja M, van Valkengoed Irene GM, Vanderschueren Dirk, Vanuzzo Diego, Vatten Lars, Vega Tomas, Veidebaum Toomas, Velasquez-Melendez Gustavo, Velika Biruta, Veronesi Giovanni, Verschuren WM Monique, Victora Cesar G, Viegi Giovanni, Viet Lucie, Viikari-Juntura Eira, Vineis Paolo, Vioque Jesus, Virtanen Jyrki K, Visvikis-Siest Sophie, Viswanathan Bharathi, Vlasoff Tiina, Vollenweider Peter, Völzke Henry, Voutilainen Sari, Vrijheid Martine, Wade Alisha N, Wagner Aline, Waldhör Thomas, Walton Janette, Bebakar Wan Mohamad Wan, Mohamud Wan Nazaimoon Wan, Wanderley Rildo S, Wang Ming-Dong, Wang Qian, Wang Ya Xing, Wang Ying-Wei, Wannamethee S Goya, Wareham Nicholas, Weber Adelheid, Wedderkopp Niels, Weerasekera Deepa, Whincup Peter H, Widhalm Kurt, Widyahening Indah S, Wiecek Andrzej, Wijga Alet H, Wilks Rainford J, Willeit Johann, Willeit Peter, Wilsgaard Tom, Wojtyniak Bogdan, Wong-McClure Roy A, Wong Justin YY, Wong Jyh Eiin, Wong Tien Yin, Woo Jean, Woodward Mark, Wu Frederick C, Wu Jianfeng, Wu Shouling, Xu Haiquan, Xu Liang, Yamborisut Uruwan, Yan Weili, Yang Xiaoguang, Yardim Nazan, Ye Xingwang, Yiallouros Panayiotis K, Yngve Agneta, Yoshihara Akihiro, You Qi Sheng, Younger-Coleman Novie O, Yusoff Faudzi, Yusoff Muhammad Fadhli M, Zaccagni Luciana, Zafiropulos Vassilis, Zainuddin Ahmad A, Zambon Sabina, Zampelas Antonis, Zamrazilová Hana, Zdrojewski Tomasz, Zeng Yi, Zhao Dong, Zhao Wenhua, Zheng Wei, Zheng Yingfeng, Zholdin Bekbolat, Zhou Maigeng, Zhu Dan, Zhussupov Baurzhan, Zimmermann Esther, Cisneros Julio Zuñiga, Bentham James, Di Cesare Mariachiara, Bilano Ver, Bixby Honor, Zhou Bin, Stevens Gretchen A, Riley Leanne M, Taddei Cristina, Hajifathalian Kaveh, Lu Yuan, Savin Stefan, Cowan Melanie J, Paciorek Christopher J, Chirita-Emandi Adela, Hayes Alison J, Katz Joanne, Kelishadi Roya, Kengne Andre Pascal, Khang Young-Ho, Laxmaiah Avula, Li Yanping, Ma Jun, Miranda J Jaime, Mostafa Aya, Neovius Martin, Padez Cristina, Rampal Lekhraj, Zhu Aubrianna, Bennett James E, Danaei Goodarz, Bhutta Zulfiqar A, Ezzati Majid (2017). Worldwide trends in body-mass index, underweight, overweight, and obesity from 1975 to 2016: a pooled analysis of 2416 population-based measurement studies in 128·9 million children, adolescents, and adults. The Lancet.

[5] Jacobsen B. K., Aars N. A. (2015). Changes in body mass index and the prevalence of obesity during 1994-2008: repeated cross-sectional surveys and longitudinal analyses. The Tromso Study. BMJ Open.

[6] Midthjell K, Lee CM, Langhammer A, Krokstad S, Holmen TL, Hveem K (2013). Trends in overweight and obesity over 22 years in a large adult population: the HUNT study. Clin Obes.

[7] Norwegian Institute of Public Health. Public Health Report: Health Status in Norway 2018. Oslo: Norwegian Institute of Public Health; 2018.

[8] Boreham C, Robson PJ, Gallagher AM, Cran GW, Savage JM, Murray LJ (2004). Tracking of physical activity, fitness, body composition and diet from adolescence to young adulthood: the young hearts project. Int J Behav Nutr Phys Act.

[9] Deforche B, Van Dyck D, Deliens T, De Bourdeaudhuij I (2015). Changes in weight, physical activity, sedentary behaviour and dietary intake during the transition to higher education: a prospective study. Int J Behav Nutr Phys Act.

[10] Reichert FF, Baptista Menezes AM, Wells JC, Carvalho Dumith S, Hallal PC (2009). Physical activity as a predictor of adolescent body fatness: a systematic review. Sports Med.

[11] Goisis Alice, Sacker Amanda, Kelly Yvonne (2015). Why are poorer children at higher risk of obesity and overweight? A UK cohort study. The European Journal of Public Health.

[12] Croezen S, Visscher TL, Ter Bogt NC, Veling ML, Haveman-Nies A (2009). Skipping breakfast, alcohol consumption and physical inactivity as risk factors for overweight and obesity in adolescents: results of the E-MOVO project. Eur J Clin Nutr.

[13] Silventoinen K, Jelenkovic A, Sund R, Hur YM, Yokoyama Y, Honda C (2016). Genetic and environmental effects on body mass index from infancy to the onset of adulthood: an individual-based pooled analysis of 45 twin cohorts participating in the COllaborative project of development of anthropometrical measures in twins (CODATwins) study. Am J Clin Nutr.

[14] Janssen I, Katzmarzyk PT, Boyce WF, Vereecken C, Mulvihill C, Roberts C (2005). Comparison of overweight and obesity prevalence in school-aged youth from 34 countries and their relationships with physical activity and dietary patterns. Obes Rev.

[15] Belcher BR, Moser RP, Dodd KW, Atienza AA, Ballard-Barbash R, Berrigan D (2015). Self-reported versus accelerometer-measured physical activity and biomarkers among NHANES youth. J Phys Act Health.

[16] Reichert FF, Wells JC, Ekelund U, Menezes AM, Victora CG, Hallal PC (2015). Prospective associations between physical activity level and body composition in adolescence: 1993 Pelotas (Brazil) birth cohort. J Phys Act Health.

[17] Kettaneh A, Oppert JM, Heude B, Deschamps V, Borys JM, Lommez A (2005). Changes in physical activity explain paradoxical relationship between baseline physical activity and adiposity changes in adolescent girls: the FLVS II study. Int J Obes.

[18] Taylor RW, Jones IE, Williams SM, Goulding A (2000). Evaluation of waist circumference, waist-to-hip ratio, and the conicity index as screening tools for high trunk fat mass, as measured by dual-energy X-ray absorptiometry, in children aged 3-19 y. Am J Clin Nutr.

[19] Kakinami L, Henderson M, Chiolero A, Cole TJ, Paradis G (2014). Identifying the best body mass index metric to assess adiposity change in children. Arch Dis Child.

[20] Ramires VV, Dumith SC, Wehrmeister FC, Hallal PC, Menezes AM, Goncalves H (2016). Physical activity throughout adolescence and body composition at 18 years: 1993 Pelotas (Brazil) birth cohort study. Int J Behav Nutr Phys Act.

[21] Baxter-Jones AD, Eisenmann JC, Mirwald RL, Faulkner RA, Bailey DA (2008). The influence of physical activity on lean mass accrual during adolescence: a longitudinal analysis. J Appl Physiol.

[22] Hallal PC, Reichert FF, Ekelund U, Dumith SC, Menezes AM, Victora CG (2012). Bidirectional cross-sectional and prospective associations between physical activity and body composition in adolescence: birth cohort study. J Sports Sci.

[23] Winther A., Ahmed L. A., Furberg A.-S., Grimnes G., Jorde R., Nilsen O. A., Dennison E., Emaus N. (2015). Leisure time computer use and adolescent bone health--findings from the Tromso Study, Fit Futures: a cross-sectional study. BMJ Open.

[24] Cole TJ, Bellizzi MC, Flegal KM, Dietz WH (2000). Establishing a standard definition for child overweight and obesity worldwide: international survey. BMJ..

[25] Cole TJ, Flegal KM, Nicholls D, Jackson AA (2007). Body mass index cut offs to define thinness in children and adolescents: international survey. BMJ.

[26] Booth ML, Okely AD, Chey T, Bauman A (2001). The reliability and validity of the physical activity questions in the WHO health behaviour in schoolchildren (HBSC) survey: a population study. Br J Sports Med.

[27] Rangul V, Holmen TL, Bauman A, Bratberg GH, Kurtze N, Midthjell K (2011). Factors predicting changes in physical activity through adolescence: the young-HUNT study. J Adolesc Health.

[28] Hermansen R, Broderstad AR, Jacobsen BK, Mahonen M, Wilsgaard T, Morseth B (2018). The impact of changes in leisure time physical activity on changes in cardiovascular risk factors: results from the Finnmark 3 study and SAMINOR 1, 1987-2003. Int J Circumpolar Health.

[29] Ekelund U, Hildebrand M, Collings PJ (2014). Physical activity, sedentary time and adiposity during the first two decades of life. Proc Nutr Soc.

[30] Wolfe RR (2006). The underappreciated role of muscle in health and disease. Am J Clin Nutr.

[31] Must A, Tybor DJ (2005). Physical activity and sedentary behavior: a review of longitudinal studies of weight and adiposity in youth. Int J Obes.

[32] Collings PJ, Wijndaele K, Corder K, Westgate K, Ridgway CL, Sharp SJ (2016). Objectively measured physical activity and longitudinal changes in adolescent body fatness: an observational cohort study. Pediatr Obes.

[33] Dumith SC, Gigante DP, Domingues MR, Kohl HW (2011). Physical activity change during adolescence: a systematic review and a pooled analysis. Int J Epidemiol.

[34] O'Loughlin J, Gray-Donald K, Paradis G, Meshefedjian G (2000). One- and two-year predictors of excess weight gain among elementary schoolchildren in multiethnic, low-income, inner-city neighborhoods. Am J Epidemiol.

[35] Basterfield L, Reilly JK, Pearce MS, Parkinson KN, Adamson AJ, Reilly JJ (2015). Longitudinal associations between sports participation, body composition and physical activity from childhood to adolescence. J Sci Med Sport.

[36] Laxer RE, Cooke M, Dubin JA, Brownson RC, Chaurasia A, Leatherdale ST (2018). Behavioural patterns only predict concurrent BMI status and not BMI trajectories in a sample of youth in Ontario. PLoS One.

[37] Wilks DC, Besson H, Lindroos AK, Ekelund U (2011). Objectively measured physical activity and obesity prevention in children, adolescents and adults: a systematic review of prospective studies. Obes Rev.

[38] van Sluijs EM, Sharp SJ, Ambrosini GL, Cassidy A, Griffin SJ, Ekelund U (2016). The independent prospective associations of activity intensity and dietary energy density with adiposity in young adolescents. Br J Nutr.

[39] Deere K, Sayers A, Davey Smith G, Rittweger J, Tobias JH (2012). High impact activity is related to lean but not fat mass: findings from a population-based study in adolescents. Int J Epidemiol.

[40] Friestad C, Klepp KI (2006). Socioeconomic status and health behaviour patterns through adolescence: results from a prospective cohort study in Norway. Eur J Pub Health.

[41] Hagquist CE (2007). Health inequalities among adolescents: the impact of academic orientation and parents' education. Eur J Pub Health.

